# Understanding the mechanisms involved in implementation of a workplace compassionate care intervention: a novel agency-structure approach

**DOI:** 10.3389/frhs.2026.1737019

**Published:** 2026-05-15

**Authors:** Cindy Faith Brooks, Jane Frankland, Michelle Myall

**Affiliations:** 1 School of Health Sciences, University of Southampton, Southampton, United Kingdom; 2 Research Innovation Services, Public Policy Unit, University of Southampton, Southampton, United Kingdom; 3 NIHR ARC Wessex, University of Southampton, Southampton, United Kingdom

**Keywords:** agency, compassionate care, England, implementation, national health service, normalisation process theory, structuration theory, structure

## Abstract

**Introduction:**

An inextricable link between context and implementation of innovations in organisations is well established. Context is understood as a dynamic landscape encompassing evolving interactive processes characterised by interactions between human actors and actors and context. Context is multi-level and operates at the micro, meso and macro. This paper introduces a novel theoretically informed agency-structure approach which was used to analyse the implementation of a workplace learning intervention targeted at enhancing relational nursing care: the Creating Learning Environments for Compassionate Care (CLECC) intervention. This approach enabled identification, characterisation and explanation of sources of contextual variation in the implementation of this intervention, offering a vehicle to understand complex mechanisms involved in introducing changes to nursing practice.

**Methods:**

A longitudinal case study design was used to study CLECC's implementation in an English mental health setting. Data collection comprised semi-structured interviews (*n* = 12) with staff involved in the implementation of CLECC, and analysis of key documents. Structuration Theory and Normalisation Process Theory jointly informed analysis.

**Results:**

A dynamic agency-structure approach enabled identification of three empirically evidenced interrelated contexts shaping the implementation of CLECC: i. COVID-19, ii. mental health and iii. nursing. Implementation was enacted at micro, meso and macro levels through these contexts, involving a complex interplay between individuals, organisations and political processes and structures.

**Discussion:**

Our novel theoretical approach provided a valuable lens for examining the complex and dynamic implementation processes, illuminating the interplay between individual agency and structural influences across multiple levels and mechanisms through which interventions are embedded or not in organisations. The value of this combined approach can be extended, with the potential for wider relevance to implementation science as a whole, enabling a unique framework through which to map the complex relations and processes shaping implementation. Through its novel theoretical approach, this paper makes an important contribution to the implementation science literature, with insights into and explanations of the mediating role of contextual variations in implementation.

## 1 Introduction

In this paper we explain what influences organisational change when attempts are made to implement a workplace learning intervention in a healthcare provider organisation. We explore how efforts to introduce CLECC–the Creating Learning Environments for Compassionate Care intervention—into an English National Health Service (NHS) mental health trust were reliant on negotiating and navigating different contexts. The mediating role of context in successful implementation is widely acknowledged (1) and dependence on contextual factors in the adoption, integration and embedding of interventions has been evidenced at length in the implementation science literature (1–12). While numerous definitions of contexts exist, we adopt one that characterises context as the landscape where implementation occurs, encompassing roles, interactions and relationships (3) and a set of individual circumstances that influence the implementation endeavour.

Our discussion is located within the sociological concepts of structure and agency (13, 14) and informed by Normalization Process Theory (NPT) (15–17) which engage with the dynamic elements of context in the implementation process (16). To the best of our knowledge, our novel use in the analysis of Gidden's structuration theory (ST) and NPT is pioneering representing for the first-time combined application of the two theories to provide a nuanced understanding of the agency-structure dynamics of implementation. This unique approach enabled: 1) identification and characterisation of the interactions between those tasked with implementation and multiple contexts in the implementation process, and 2) explanation of the work involved in attempting to manage these contexts. A recursive relationship between agency and structure manifested as attempts were made to implement CLECC impacting the extent to which implementation was successful. The value of this combined approach can be extended yielding wider relevance to implementation science as a whole and enabling a unique framework through which to map the complex relations and processes shaping implementation.

### 1.1 Description of the intervention

CLECC is designed to restructure nursing ward-team activities in a way that seeks to compensate for a lack of relationship continuity in acute care settings, targeting workplace conditions to support compassionate care practices rather than targeting individual behaviour change (for example, techniques to address unprofessional behaviours such as rudeness or bullying (18). CLECC introduces a programme of workplace learning, led by a project facilitator to develop relational practices such as dialogue, reflection and mutual support (19). During an introductory implementation period the educator works with the team to identify which of a number of suggested activities (Table 1) are feasible and useful and to establish these within everyday practice.

**Table 1:** CLECC activities implemented.

Activity	Purpose	Frequency	Staff involvement
Cluster discussion sessions also referred informally by staff as “huddles”	5-minute team get-togethers to feedback on the ongoing shift	Weekly from the beginning to post implementation period	Led by Internal Facilitator
	Checking on staff wellbeing and providing problem solving and support		Nursing staff regularly attended the week day huddles
			Non nursing staff were invited but attendance was limited
Reflective sessions	Planned group activities to prompt personal reflection, dialogue and reflective learning	Scheduled twice weekly during initial months of the implementation period	Led by the Internal Facilitator and mainly involving nursing staff
Peer observations	A structured observation method to present a review of positive care to the team for discussion and reflection	Scheduled irregularly in the first few months of the implementation period	Internal Facilitator involvement with nursing staff
Team introductory sessions	Introductory learning sessions about the purpose of CLECC run as a whole team via a team study day or away day or multiple shorter sessions	Scheduled in the first month of the implementation period	Both Internal and External Facilitator involved in leading the sessions
		Primarily nursing staff attended, with limited participation from other professional groups such as medical staff	
CLECC sustainability plan	To take CLECC forward over the subsequent 12 months	Completed once towards the end of the implementation period	Internal Facilitator and ward management team

A mixed-methods feasibility study of CLECC was conducted in two general acute care hospitals and completed in 2016 (20). Study findings showed that CLECC's degree of impact and sustainability were highly context-specific and mediated by factors both at ward-team level and other levels of the organisation (21). This highlighted the need for further studies to better understand the scope, sources and impact of variation, including more systematic study of contextual layers beyond individual teams.

### 1.2 Theoretical framework

**Figure 1:**
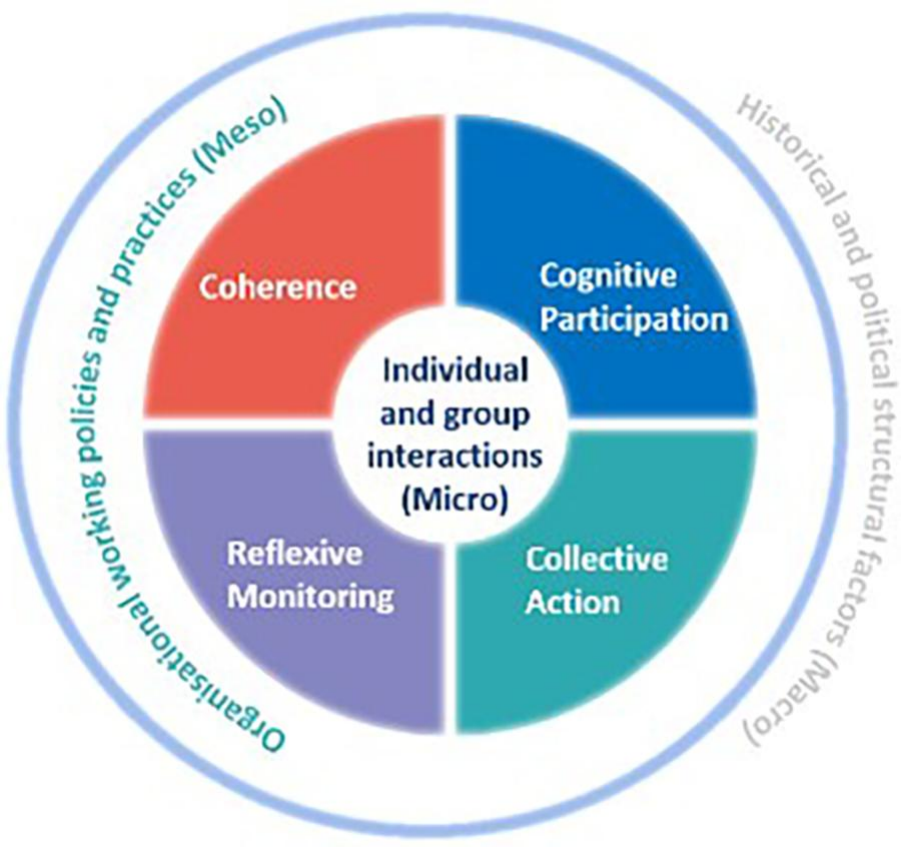
Theoretical approach: An agency-structure model-normalisation process theory with structuration theory (6, 7, 15, 22–24).

We employed a novel theoretical approach which accommodates the dynamic interplay between agency and structure, NPT and ST (6, 7, 15, 22–24) outlined in Figure 1.

NPT focuses on the micro (e.g., individual and team considerations such as coherence and participation) and meso (e.g., organisational considerations such as working policies and practices) mechanisms involved in implementation, offering a means to understand how complex interventions are a product of interactions between the properties of the intervention, the collective action of its users, and the contexts of its use (15, 16). In this way, agency, is constituted within these micro-meso relations, between individual and group interactions with organisational practices and policies. NPT effectively characterises mechanisms that motivate and shape processes leading to translational successes and failures and influence the extent of implementation. These mechanisms are coherence—making sense of the intervention; cognitive participation—investing in the intervention; collective action—the practical work of implementation; and reflexive monitoring—modifying and embedding the intervention (15, 16).

Whilst NPT examines the micro and meso considerations of implementation, ST focuses upon the structural (macro) levels. Where agency features in NPT in the micro-meso relations between individual and group interactions with organisational policies and practices, ST extends understanding of agency to consider interactions with macro considerations (e.g., structural elements such as historical and political factors), as well as the dynamic interplay between agency and structure throughout these dimensions. For Giddens, agency is determined by human action, which constitutes, sustains and shapes structure (14, 25). Neither agency nor structure are afforded precedence, but are interlinked, shaping one another (13, 14). Giddens conceptualises this co-dependence between agency and structure as a “Duality of structure” (13, 14).

Combining NPT with ST, directly addresses the call to utilise NPT as a “conceptual toolkit” to be used independently or in association with other theories (22), enabling a whole system approach to implementation, whereby the influence of implementation processes are examined at micro (individual), meso (organisational) and macro (healthcare) levels (22). Moreover, combining theories can help to attend to the specific aims of an implementation project (26) in this case facilitating understanding how agency-structure dynamics are involved in the implementation of CLECC at micro, meso and macro levels.

### 1.3 Aims and objectives

To identify, characterise and explain sources of contextual variation in the implementation of CLECC and to better understand the complex mechanisms involved in implementation.

Specific objectives:

- Describe the contextual variations of implementing CLECC in a mental health in-patient care setting
- Identify and characterise how agency-structure dynamics are enacted through these contextual variations
- Explain the micro, meso and macro features of agency-structure dynamics through contextual variations

## 2 Methodology

### 2.1 Study design

The study employed an in-depth mixed-methods longitudinal case study design (27). Case study was chosen to capture the impact of variations over time, in target teams and within the wider organisation, and to build a picture of longer-term sustainability. We used semi-structured interviews and document analysis to investigate real-time CLECC implementation. Using a case study design yields insights into the “how, what and why” of the implementation of changes to practice (28) and exploration of social, political and economic contexts (29). We define our approach to case study as exploratory; and case refers to the processes involved in implementing CLECC in a mental health setting.

### 2.2 Ethical approval

Ethical approval was obtained from the University of Southampton Ethics Committee (Reference: 57037), and governance approvals from the UK's Health Research Authority, and the participating NHS Trust. Informed consent was received from all participants.

### 2.3 Setting and participants

The study was conducted in an inpatient ward in an NHS mental health Trust in England between 2020 and 2022. The ward was composed of mainly nursing staff from Band 2 (e.g., Healthcare Support Worker) to Band 8a (e.g., Matron), with some health care support staff and staff from other occupations (e.g., doctors and physiotherapists).

The facilitation of CLECC's implementation continued for 18 months from October 2020 and remained active at the time of follow-up interviews in early 2022. Purposive sampling was used to identify participants representing a variety of roles within the Trust including Health Care Support Workers (HCSWs) (*n* = 3), Mental Health Nurses (*n* = 3 including 1 at a senior level), Staff Nurse (1), Senior Manager (1), an Internal CLECC facilitator (1) and External facilitator (1). The Internal Facilitator was responsible for the day-to-day CLECC facilitation, and the External Facilitator provided an advisory role on CLECC's implementation and coaching to the Internal Facilitator.

### 2.4 Interviews

Semi-structured interviews (*n* = 12) over Microsoft Teams were undertaken by the first author at two time-points. Ten were conducted three or more months following the start of implementation, (January 2021 to May 2021), and follow-up interviews were also held with the Internal Facilitator and External Facilitator (January 2022 to February 2022). Interviews were digitally recorded and lasted 30–60 min and included questions that addressed understanding of CLECC and perceived value.

### 2.5 Documentary evidence

Documentary evidence consisted of two types. First, CLECC specific data collection documents which included: details of attendance at planned CLECC learning activities (completed by the Internal Facilitator); CLECC Internal Facilitator fieldnotes, intended to record observations about implementation; CLECC sustainability plan (developed as part of project implementation); a ward profile form to collect core information about the ward (e.g., size, staffing, leadership, staff absence and patient feedback) completed in April 2021 and again in December 2021 by a ward leader. Second, a range of Trust level policy documents were collected, e.g., clinical supervision policy, therapeutic observations and engagement policy (Table 2).

**Table 2:** Documents included in analysis.

Document name	Date of document
CLECC Internal facilitator field notes	2/2021
Facilitator Huddle attendance numbers	2/2021
Facilitator report on attendance at CLECC learning activities	2/2021
CLECC Sustainability Plan	1/2021
Ward Profile Form	4/2021
Ward Profile Form	10/2021
Trust Band 5 Induction Information for ward—Part 1	9/2018
Trust Band 5 Induction Information for ward—Part 2	9/2018
Trust Bank Agency Induction forward—	8/2020
Trust Therapeutic Observations and Engagement Policy	12/2020
Trust Strategy	3/2020
Trust Clinical Supervision Policy	10/2020
Trust vision poster	2020
Trust Freedom to Speak Up Policy	3/2020
Trust Improving and Managing Conduct Policy	12/2020
Trust Induction and Essential Training Policy	9/2019
Trust Learning and Development Policy	9/2018
Trust Managing Attendance and Wellbeing Policy	3/2020
Trust Managing Stress at Work Policy	7/2018
Trust Performance Management Policy	3/2020
Trust Recruitment and Selection Policy	12/2020
Trust infection-prevention-control-policy	3/2022
Trust response to COVID-19, plans for restore and recovery approaches.	6/2020
Trust Quality Account 2018/19	No date

### 2.6 Data analysis

Interviews were transcribed verbatim by a professional transcriber and analysed by the first author. Findings were discussed in data workshops with the research team to identify patterns and connections across themes and in team meetings where interpretations were discussed and tested out. Documents were analysed following interview data analysis. Documentary data were used to contextualise and triangulate interview findings.

Analysis was theoretically informed by our agency-structure approach. A six-stage approach to thematic analysis involving development of an inductive coding frame was used (30):

(i) Familiarisation; reading and re-reading the interview transcripts to become familiar with the data
(ii) Generation of coding categories
(iii) Grouping of codes into contextual themes
(iv) Mapping how these contexts relate to the theoretical framework (i.e., each NPT mechanism and agency and structure dynamic)
(v) Naming of the themes or contexts (e.g., COVID-19, mental health or nursing)
(vi) Presenting the thematic framework for the data through discussion in data workshops with the research team to identify patterns and connections across themes and in team meetings where interpretations were tested out

This approach utilises a combination of a more structured approach to coding with reflexivity (30), which was enacted through regular research team meetings (people with backgrounds in nursing and sociology) and collective substantial clinical and/or research experience across health and social care settings). Themes were generated from interview data using these steps and in accordance with the theoretical approach outlined above, to enable understanding of how agency-structure dynamics are enacted through the interaction of NPT mechanisms in accordance with micro, meso and macro features.

A similar team-based discussion approach was adopted for the documentary analysis undertaken by the second author. CLECC specific qualitative documents were analysed for data relating to codes and contexts identified from the interviews and to consider the existence of additional contexts. No new contexts were identified in the documentary data. CLECC documents were used to support the main interview analysis and NHS Trust policy documents were used to contextualise the findings from the qualitative analysis (Table 2).

## 3 Results

Three interrelated thematic contexts were identified pertaining to CLECC implementation: 1) COVID-19, 2) mental health, and 3) nursing. Each context is discussed in relation to how participants enact agency between the micro (individual), meso (organisational) and macro (political) structures involved. Quotations and documentary extracts demonstrate the dynamism between agency and structure.

### 3.1 COVID-19 context

This context refers to the ways in which COVID-19 influenced and shaped implementation of CLECC in the Trust. Implementation coincided in England with two national lockdowns, government policy on the use of Personal Protective Equipment (PPE) in health care settings, mandatory social distancing, and a requirement for 10 days of self-isolation following contact with an individual who presented as a positive COVID-19 case (31). For those working in mental health settings, the impact of COVID-19 and associated national policies enacted at a macro level presented unique challenges and concerns for those providing (safe) inpatient care at an organisational or meso level, with national guidance identified elsewhere as confusing, constantly changing and lacking sufficient specificity for the setting (32, 33). In the context of this uncertainty, staff involvement in CLECC activities occupied a tenuous role between offering opportunities for individual and group support operating at a micro level whilst presenting challenges through risk of exposure to COVID-19 or of breaking social distancing guidelines.

Participants perceived purpose and value (coherence) in CLECC activities and events, in particular the huddles and introductory sessions (Table 1) were seen to offer emotional and professional support mechanisms to individuals and teams in relation to COVID-19. First, at a micro level participation provided an opportunity for staff to share their anxieties and gain mutual reassurance from colleagues and management in response to COVID-19. This communal support strengthened team relations and boosted staff morale. Second, CLECC activities served as a source of information gathering and learning about how to work and manage uncertainties in response to shifting COVID-19 organisational and national guidelines and policy including those related to infection control, isolation, and PPE:

COVID rules are changing always on the ward. PPE, visiting, swabs, isolation and we've got to the point where it's sometimes hard to really keep up … So, the huddles-if we don't know what's going on with the isolation-we got really confused … if you've got any worries or concerns you can express it without any judgement and get the right answers there and then. (HCSW 1)

I think, a lot of people really threw themselves into [huddles], it's so nice to just be able to have a chat together and just be like “actually, I am really fed-up of COVID”, “I'm really fed-up of the ever-changing rules on the ward” and it's brought us together as a team a lot more. (HCSW 2)

While CLECC was viewed as a valuable intervention for providing emotional and professional support to individuals and teams, concerns about the appropriateness of engaging and participating (cognitive participation) in CLECC activities, in accordance with organisational and government policy on social distancing in response to the infection risk associated with COVID-19 were reported. “The huddle” activity, which required staff to congregate in a confined space, was perceived as potentially problematic as it increased time spent in close proximity, risking cross-infection. Ensuring staff adhered to organisational COVID-19 infection policy and social distancing guidelines was a priority for managers. This limited opportunities for CLECC implementation from a strategic perspective:

So, we had an outbreak of COVID on the ward at the end of January, and we felt it was more appropriate to not have the daily huddles because we shouldn't be having a lot of staff all in a small space for a long period. (Senior mental health nurse 2)

COVID stuff was the priority … that was what was on everyone's minds and also management getting that right and making sure everyone understood those procedures … there wasn't a lot of headspaces for [CLECC] as well, it was very much an add-on. (Internal facilitator)

The introduction of CLECC occurred when COVID-19 and associated policies and guidance was significantly impacting on work practices and influenced staff involvement (collective action) in CLECC activities. The introduction of new COVID-19 related organisational policy and guidance, enacted at a meso-macro level, combined with high levels of staff sickness absence, the need to isolate, and re-deployment requirements to meet additional workforce needs, directly shaped individual and team dynamics operating at a micro level, creating unprecedented pressures on staff and their ability to undertake routine work practices. Consequently, CLECC activities were sometimes postponed or cancelled:

Introductory [CLECC] session was planned; however, I received an email from [the] ward secretary yesterday requesting that the slot be given to Infection Prevention Control to deliver an emergency session on IPC [Infection Prevention and Control] processes and PPE (Internal Facilitator Fieldnotes February 2021, entry dated 09/10/2020)

Conversely, from a senior management perspective it was perceived, through informal conversations with staff, that CLECC participation did not increase staff workload during the early stages of COVID-19:

[CLECC] wasn't seen as a burden even at a time when it could have been seen as something else to do while we were sort of trying to—because it was early COVID stage, wasn't it? (Senior manager)

Follow-up interviews highlighted several issues associated with the prioritisation and impact of COVID-19 which influenced the continued involvement (reflexive monitoring) of staff in CLECC. Key barriers were identified as staff shortages due to sickness absence because of contracting the virus and isolating; adherence to organisational infection control policies operating at a meso level; and prioritisation of national COVID-19 programmes at a macro level impacting upon the time and capacity of senior leadership staff who were actively involved and supported CLECC:

I think [COVID] had a massive impact to be honest. So, there were several periods where there were quite large numbers of staff isolating, so then shifts were being covered by more bank nurses and agency workers … the very practical thing of having huddles where people are grouped together is not particularly advised during COVID and I think infection control [were] maybe not too happy about that sort of thing … it really did hinder the sustainability of the project. (Internal facilitator)

We started this at the time of COVID when we're also trying to step up some large-scale vaccination centres … Had that not happened, we would have also had [a senior nurse manager] … in the steering groups. (Mental health nurse 1)

Findings suggest that while staff were not resistant to the idea of participating in CLECC, there were questions about how its implementation could be supported during COVID-19. These related to how continuity could be supported in the face of staff shortages, resulting increased workload, and the appropriateness of CLECC activities in relation to organisational policies and guidance regarding pandemic measures (e.g., infection control policies or social distancing). In addition, a key challenge was navigating the complex and often inconsistent organisational policy and guidance to manage risks associated with COVID-19:

So, I think, ideas about risk are problematic … , the same guidance can be interpreted hugely differently by different organisations, and this was particularly complex … there was a lot of stakeholders … and I think, that had an impact about speed and ability to get into organisations and work with organisations even, given the pandemic. (External facilitator)

Other enabling factors relating specifically to CLECC implementation during COVID-19 included provision and availability of organisational resources enacted at a meso level to support hybrid or virtual working. At a micro-level provision of organisational resource actively influenced individual and team involvement in CLECC activities as each staff member would require access to their own computer/laptop and adequate space to participate. In addition, for facilitation of hybrid or virtual interaction working, the provision of essential resources, and an opportunity to claim back time for involvement were limited:

[being online] probably extended [their] working day … so, from her (Internal Facilitator's) point of view, they chose the times that we met, and they happened to be when they were at home and not on the unit. From my point of view, it helped because they were able to engage. They weren't being pulled off into doing clinical work so, sessions started and finished on time, and I had their full attention during the sessions … also at home [they] (Internal Facilitator) had access to a computer whereas, in that unit it was clear from when we tried to do the study day that everybody didn't have individual access to a computer. (External facilitator)

I mean you could [be] online but it's quite a fatiguing thing to do a whole day online, you don't get that same interaction because you can't really have more than two people speaking at the same time. You could have breakout groups but again that's quite hard to organise and oversee … but I personally think that you lose engagement with big group things when they're done online …. I suppose the other way of doing it would have been to do more days and have less staff in the room so you could socially distance … but at the time it just wasn't practical with rooms that were available and shift patterns. (Internal facilitator)

### 3.2 Mental health context

This context relates to the way in which implementation of CLECC was impacted by macro and meso level policies and working practices relating to mental health. In the context of these influences, implementation of CLECC occurred mainly at a micro level, providing opportunities to support the mental health of staff as well as to reflect upon and resolve patients concerns. The purpose and value (coherence) of CLECC in a mental health setting was recognised. While there was similarity with some existing staff support mechanisms in this organisational setting, staff reported added value from CLECC activities. In particular, the huddles and reflective sessions were considered to provide staff with tools within an explicit structure and framework to come together collectively to discuss and reflect on patients' needs:

in most mental health settings, a well-established aspect of working in those settings is having one-to-one supervision and participating in reflective practice sessions and those things are, you know, very similar to some of the suggested interventions in [CLECC]. So, I think, there is a risk that people see: “well, what's the extra value then?”. But I do think there is an extra value because, even though these things [one-to-one supervision and participating in reflective practice sessions] are more firmly embedded, there may be an absence of clear intention around why we're doing these things—and what we're hoping to get out of them. (External facilitator)

In the context of these organisational pressures engagement (cognitive participation) in “huddles” and “reflective sessions”, provided support for staff wellbeing and helped alleviate some of the pressures of working in a mental health in-patient unit. At a micro level, through individual and team engagement, CLECC offered staff time away from the ward to reflect and share issues or concerns, though it was recognised that this opportunity was dependent upon organisational provisional of appropriate equipment and adequate resource to facilitate their involvement:

there was not enough—magnets for the beds—because obviously our patients are “mental health”. Sometimes we have to lock the remote control on the bed because they can play with them, going up and down, and, I think, they were ordered … more keys and more magnets so that all staff would have some. It's just little things like that. (HCSW 3)

sometimes working in a mental health environment, don't know which word to use but if staff could have just that time. Not just break time—just that time away to talk to each other about things they're struggling … that will be helpful. (Mental health nurse 2)

Participation in huddles was recognised as having a positive impact upon existing working practices in regard to supporting patient care, by enabling staff to raise and resolve both patients' and their own concerns, as well as serving as a reminder about compassionate care when returning to the ward.

it's like if patients do have a concern, you can maybe raise it to the staff and if you're concerned about it, you can raise it in [CLECC] huddles, and it gets sorted, and [you] get a voice and then you can help the patient. I find it really nice. (HCSW 1)

Because I think with staff talking to each other, bringing up any issues, it helps with stress and anxiety-if you're stressed and anxious, that can be passed on to the patients which makes them anxious … So, yeah, I think, a happy ward equals happy patients. (Staff nurse)

However, a perceived barrier to participating in CLECC activities in relation to existing working practices (collective action) was the heightened level of supervision and care required in a mental health ward compared to a general ward, related to the theme of COVID-19 The Trust's Therapeutic Observations and Engagement Policy (December 2020), sets out the principles and standards for patient observations within inpatient services. The policy requires high levels of observation for at-risk patients, including keeping those at imminent and high-level risk of harming themselves or others within eyesight and arm's length. In line with this, participants reported that leaving patients unsupervised to take part in activities could result in potential risk. Moreover, for some staff, time away from their ward to undertake CLECC activities heightened anxiety and concerns stemming from not being available for patients and placing additional burden on colleagues who were left to manage in their absence:

there is no kind of one time where all staff are going to be available and able to participate. So, it's trying to kind of balance the risks of making sure that the ward is safely staffed while we're holding those huddles. (Senior Mental health nurse)

Although I was covering the HCSW doing the peer observation, she appeared anxious about not responding to patients' requests, and half-way through abandoned the observation to help another HCSW with a restless patient, despite there being other staff in the area. We discussed this together afterwards, and she confirmed she didn't feel comfortable not helping out. (Internal Facilitator fieldnotes February 2021, entry dated 06/11/2020)

Whereas another participant did not think working in a mental health ward posed specific barriers to participating in CLECC activities, instead seeing workload and lack of priority as a main barrier to participation:

I can't think of any particular barriers which are specific to mental health… the barrier is the busyness and people losing its importance. That isn't specific to mental health. That could be in any environment … Loss of priority because it can slip down … people's importance, can't it? (Mental health nurse 1)

While participants identified both barriers and facilitators (reflexive monitoring) to implementing CLECC in a mental health in-patient ward, there was a level of doubt regarding longer term sustainability and becoming “business as usual”. This was primarily attributed to challenges of accommodating the various needs of different types of staff and prioritising the specific and complex needs of patients within a mental health setting:

Finding a time when everyone (e.g., occupational therapists, physios, support workers, doctors, kitchen staff, domestic staff etc…) can participate in the huddles was difficult. (Senior mental health nurse)

People will want to make [CLECC] a priority, to carry them on but it's just at some points if you take even like one or two staff members away for five minutes, it puts patients at risk so, we're just not able to do it. (HCSW 2)

A key output from CLECC was for the Internal Facilitator to support the ward management team in the development of a sustainability plan. This addressed the impact of increased surveillance levels required on mental health wards on the ability to undertake CLECC peer observation activity, with recognition that cover may be needed to enable staff to participate. There was acknowledgement of the need for forward planning for this activity to be sustained:

Depending on the patient acuity level and staff-to-patient ratio at the time, staff may need to be covered for the hour of their observation period. They will book in their observation ahead of time and liaise with the ward manager, senior nurses, or other staff on management days, who could provide this hour's cover. The staff member will also write this in the ward diary, so the Nurse in Charge is aware on the day. (Sustainability Plan, January 2021)

Interview data suggested that leadership of the ongoing facilitation of CLECC, required key qualities such as an interest in staff wellbeing and experience in facilitation:

if you're just interested in supporting staff wellbeing and understanding how to facilitate groups, I think, you would be perfectly well placed. (Internal facilitator)

### 3.3 Nursing context

The context of nursing relates to the way CLECC was generally perceived by staff and how this perception shaped different professional groups' participation in CLECC activities, enacted at a micro level, as well as recommendations for the sustainability of CLECC through nursing leadership acknowledging the need for senior organisational buy-in and adequate resources and support.

Participants generally perceived the purpose (coherence) of CLECC as a nurse-led initiative designed to enhance compassionate care. However, staff from a range of different occupational backgrounds were encouraged to attend CLECC activities. Many participants recognised that the underlying principles of compassionate care extended beyond nursing and were valuable for the practice of other professions within the organisation and CLECC activities were intended to provide opportunities for staff to develop their occupational roles by sharing expertise and knowledge across disciplines. Nevertheless, the purpose and relevance of CLECC activities was not always fully understood, and some professional groups viewed the activities as unnecessary:

Compassionate care doesn't just come from the nursing staff, does it? Or the medical staff or the therapy staff. The people which have the most contact with patients, it's probably the cleaners … whereas nursing staff are speaking to one person, or a group of people for a period of time. The cleaners are there all the time. (Mental health nurse 1)

at the beginning they wanted all the staff groups to attend including non-clinical staff like the kitchen staff and the domestic staff but that was really tricky. I think, the domestic staff didn't really understand why they were being asked to join. (Internal facilitator)

I emphasised the importance of the therapies team sharing their expertise in the reflective sessions. The group felt that although they understood the idea of the huddles, they did not benefit from these as much as other staff as they are already very good at debriefing with one another on a daily basis. (Internal Facilitator fieldnotes February 2021, entry dated 07/12/2020)

Although staff from different occupational roles were encouraged to recognise the value of CLECC to their practice, with many being invited to participate in CLECC activities (cognitive participation), attendance predominantly comprised nursing staff and HCSWs.

it's usually done within a skills—in-between each handover. So, it can be nursing staff, support workers. I think we had some occupational therapists in there at one point, the manager, came on a few as well. (Staff nurse)

It is worth noting that there were no senior nurses or managers present, and no medics or Allied Health Professionals, as they were unable to attend. (Internal Facilitator Fieldnotes February 2021, entry dated 9/11/2020)

Accommodating CLECC activities within daily working routines, particularly the responsibility for embedding related activities without the required support, was identified as a barrier to participation by non-nursing staff:

at times it's been a bit of a challenge … that's not what they're used to doing. (Mental health nurse 1)

Non-nursing staff were invited to the huddles—although some were able to attend sometimes, many found the huddles did not fit in with their work patterns. (Facilitator report on attendance at CLECC learning activities)

Participation in CLECC activities (collective action) appeared to contribute to the wellbeing of nursing and HCSW staff, through enhanced relational working between nurses and their HCSW colleagues, affording increased insights into each other's roles. In addition, the role of CLECC activities such as the huddle and reflective sessions provided a structured support mechanism, allowing nurses and HCSWs to reflect on how their aspirations or care aligned with wider organisational expectations of care, whilst acknowledging specific concerns staff had about attending sessions in accordance to organisational and government policy in association with the infection risk associated with COVID-19, as well as anxieties in attending the activities, and leaving patients unsupervised, discussed in the earlier themes of COVID-19 and Mental Health. This reflective engagement was viewed as contributing to improvements in the quality of patient care:

Yeah, I think, [the reflective sessions] were very good. They were really useful to get everyone's point of view across … Definitely like between nursing staff and support workers … I think, the relationship between nurses and support workers has got better personally. (Staff nurse)

it introduces people to a set of easily learnt practical tools and means of encouraging conversations about their own wellbeing and how they as individual practitioners are. Like the huddles which is really, really helpful … , it gives a structure, some tools and some facilitation to enable people to think more deeply about themselves and the care that they give as individuals and a team. (External facilitator)

Participants suggested that sustained use of CLECC (reflexive monitoring) would require a number of resources to be provided by the organisation including: a dedicated “nursing role” with routinised time and space to facilitate the delivery of CLECC, with consideration given to whether the person appointed needed to be internal/external to the organisation, their grade and position, their personal approach and commitment to the role, and being well-respected by others:

taking that leadership role, a well-respected clinician … so, [name of Internal Facilitator] quietly confident. Very, very capable … (Senior manager)

yeah, [the Internal Facilitator] just took it and ran with it … was absolutely fantastic so, it didn't need as much support from the leadership team as … just kind of jumped on-board with it. (Mental health nurse 2)

Participants emphasised the value of the individual (micro level) characteristics associated with the democratic and inclusive leadership style of the Internal Facilitator. Their “quiet leadership” style, evident passion for the role, and status as a respected existing employee of the Trust were viewed as key enablers for engagement with CLECC. Participants also emphasised that the long-term sustainability of CLECC depended on organisational commitment, including support from senior nursing leaders at ward and executive levels and the provision of dedicated resources, ensuring that the facilitator role was not reliant on voluntary effort:

 it would need to be matron and the ward manager, needs to maintain its—importance. So, they continue to put aside a time for in their skills slots and having (the Internal Facilitator's) time. (Mental health nurse 1)

A sustainability plan (Sustainability Plan, January 2021) was developed at the end of the three-month period of implementation, in consultation with the leadership team and the ward staff, to document how continued commitment to CLECC activities would be achieved. This document illustrates that CLECC activities will be led by nursing staff, but stresses that all staff (clinical and non-clinical) are invited to attend. One member of nursing staff would co-ordinate the daily huddles, including inviting any non-nursing staff present on the ward at the time. Beyond this, attendance of non-nursing staff was left to individuals.

One follow-up interview indicated that the huddles had continued briefly after the implementation period. A combination of organisational factors, including the need for sustained senior organisational support, insufficient resource to continue support of the Internal Facilitator role, as well as a change in the ward manager had contributed to the discontinuation of CLECC activities.

## 4 Discussion

This paper provides a novel theoretically informed agency-structure approach to identifying, characterising and explaining sources of contextual variation in the implementation of a workplace intervention in a mental health setting. The CLECC intervention has been used as a vehicle to better understand the complex mechanisms involved in implementation and, specifically, to identify and characterise how agency-structure dynamics are enacted through these contextual variations and to explain the micro, meso and macro features of these dynamics.

Combining Structuration Theory and Normalisation Process Theory within a case study design provides valuable understanding into variations of implementation through three interrelated thematic contexts; COVID-19, mental health, and nursing. The discussion integrates these findings with existing research to provide a comprehensive understanding of the implementation of CLECC in these contexts.

### 4.1 Integration with existing research

#### 4.1.1 COVID-19 context

CLECC was implemented from October 2020 at the height of COVID-19 at a time when health and social care systems faced unprecedented pressures (31). Hospital staff had to learn to work in different ways and for long hours in challenging circumstances (34) and reconfiguration of services meant some staff were redeployed to other clinical areas to cope with increased demand. For those working in mental health, there was frustration about changing advice during the pandemic and COVID-19 guidance was viewed as confusing, and lacking specificity for the setting (32, 33).

In this study setting, COVID-19 had a significant impact on CLECC implementation. Staff had to adapt in accordance with a context of unprecedented change (35–40) and whilst facing heightened emotional and physical demands requiring strategies for resilience (41). The height of COVID-19 and associated policy resulted in macro and meso level intervention in healthcare settings, in terms of guidance for ways of working, including social distancing and isolation, and demands for redeployment of staff. This had implications at a micro level for staff participation, meaning that, in addition to the common challenge of finding time for huddles (21), staff also had to navigate these rules preventing staff coming together in groups. This led to both uncertainty and conflict for staff, with CLECC offering a mechanism for support but simultaneously presenting risk in terms of compromising social distancing guidance and creating potential exposure to COVID-19. Participation was further impacted by staff shortages related to COVID-19 isolation and illness, reflecting wider incidence. This prevented securing cognitive participation needed to achieve collective action around CLECC to bring about change.

In addition, sustainability of CLECC was challenged in the context of such uncertainty. Key considerations for successful CLECC implementation in the COVID-19 context included supporting continuity in the presence of staff shortages, adapting activities to comply with COVID-19 related infection control policies, ensuring adequate resources to support safe participation, and accommodating disruptions to a person's usual professional roles and routines. As these conditions could not be fully achieved for CLECC in the COVID-19 context, it was difficult to accomplish the collective action needed to fully embed and sustain the intervention beyond initial implementation.

Given recent evidence of the ongoing risks which contracting COVID-19 continues to present for the NHS workforce, in developing long COVID and other health conditions coupled with a lack of systemic support for mental health and wellbeing (42), findings underscore the importance of understanding the perceptions and experiences of staff in fulfilling their professional role in accordance to complex and highly adaptative contexts. Findings also contribute to the limited evidence on how to support frontline staff in providing compassionate care in the context of COVID-19. This underlines the need for organisational leadership and resource to support staff in the context of unprecedented pressures and associated risks such as navigating changing infection control policies (43, 44).

#### 4.1.2 Mental health context

Mental health inpatient wards can be highly stressful workplace environments, and the profound effects this has on staff wellbeing and practice (45) and patient care and outcomes (46) is widely acknowledged. To address the detrimental impact of workplace stress in mental health settings, support mechanisms for staff have been increasingly introduced as usual practice both in UK settings and internationally (47). However, there is little understanding of how these support mechanisms have been perceived by mental health staff, in particular mental health nurses (48). In the study setting, “huddles” added value to these existing mechanisms, offering an explicit structure and framework for staff both to discuss and reflect on the mental health needs of patients in their care, and articulate and navigate the personal pressures of working in a mental health environment.

However, the extent to which staff could participate in CLECC activities was influenced by macro and meso level policies, with care requirements informed by the political and regulatory context of national guidelines informing organisational adult mental health policy and Trust policy for vigilant observation of at risk-patient safety. These could be seen at odds with CLECC activities such as the daily huddle. Professional obligations had to be prioritised, which influenced sustained collective action. Taking part in CLECC activities required participants risking meeting their moral and legal obligations as mental health professionals. The specific complexities of working in a mental health setting meant that implementation included navigating a context that operated at a primarily micro level and was reliant on staff's capacity and motivation to enact CLECC activities and make potentially sustainable change to practice. For CLECC to have gained traction, the necessary resources needed to be in place, such as adequate staff provision to enable attendance as well as processes to support advanced booking of involvement in CLECC activities ahead of time.

#### 4.1.3 Nursing context

While CLECC is a nursing-led initiative, the principles of compassionate care underpinning CLECC were perceived by the team as extending beyond the needs of the nursing team and as having value for other professions. However, participation in CLECC activities was predominantly undertaken by nurses and HCSWs. The perceived value of participation was that CLECC activities provided opportunities for enhancing relational working between nursing and HCSW colleagues in the Trust.

There is evidence of active maintenance of professional boundaries within settings where multidisciplinary teams are key (49, 50) and the underrepresentation of other professions in CLECC activities could be explained by this macro-level context; insofar as CLECC was perceived as nurse led may have constrained perceived relevance to other professional groups.

A supporting factor was that the introduction of CLECC incorporated a research component, which was central to the organisational culture within the Trust. This research culture is also supported by national policy promoting nurse-led research (51). However, it was also acknowledged that sustainability of this role was only possible with meso-level buy-in and engagement from senior nursing professionals, both within the ward and across organisational senior leadership, alongside the provision of adequate resources.

### 4.2 Implications for policies and practice

An agency-structure approach affords key insights for implementation science. It has been suggested that many implementation frameworks are hierarchical, assuming a downward linear approach to implementation (52). By identifying the agency-structure dynamics involved in the implementation of CLECC, implementation is recognised as a contested space, involving navigation by individuals (micro) with organisations (meso) and wider historical and political factors (macro). By examining these interacting dimensions, it supports a whole system approach to implementation within and across these levels (22).

### 4.3 Strengths and limitations

A dynamic agency-structure approach utilising a longitudinal case study design enabled identification of the three interrelated contexts that shaped the implementation of CLECC, adding to understanding of relevant contextual layers beyond the team. The analysis of these three contexts has allowed comparison which highlights the interplay between agency and structure across micro, meso and macro levels. This study would have been enhanced by the use of direct observations of implementation efforts, but the pandemic context meant this was not possible and so our findings rely on participants' perceptions of what happened. Further research could utilise our approach in other settings to better understand variation between teams and organisations.

## 5 Conclusion

Combining NPT and ST has demonstrated how agency is enacted through micro, meso and macro interactions, providing a unique and novel lens to understanding the complexity of implementation through continual interaction and navigation between individuals, organisations and wider political and historical processes and multiple structures that make up these contexts. By adopting an innovative dynamic agency-structure approach, this paper has identified mechanisms involved in the implementation of a complex intervention to facilitate compassionate care in an NHS mental health setting. Understanding the perceptions and experiences of those involved is integral to examining the complexities of implementation and relations to wider meso and macro structures, with implementation recognised as a highly adaptive complex process, involving uncertainty and applying learning from those directly involved in navigating change, as implementation proceeds. Furthermore, this lens affords invaluable insight for the scale up of interventions such as CLECC, which have been identified as high priority by policymakers in recent years, conceptualising sustainability as a fluid space to be revisited in relation to shifting agency-structure dynamics over time.

A leading recommendation is that this original dynamic agency-structure approach has relevance, and utility for other contexts and settings involving implementation and implementation scientists, affording distinctive and nuanced insight and transferable learning into complex dynamic relations and processes.

## Data Availability

The datasets presented in this article are not readily available because of restrictions governed by the ethical agreement approved by the University of Southampton.
